# Guanidinium quinoline-2-carboxyl­ate

**DOI:** 10.1107/S1600536809049733

**Published:** 2009-11-25

**Authors:** Graham Smith, Urs D. Wermuth

**Affiliations:** a School of Physical and Chemical Sciences, Queensland University of Technology, GPO Box 2434, Brisbane, Qld 4001, Australia.

## Abstract

In the structure of the guanidinium salt of quinaldic acid, CH_6_N_3_^+^·C_10_H_6_NO_2_^−^, the asymmetric unit contains two independent cations and anions having similar inter-species hydrogen-bonding environments, which include cyclic *R*_2_^2^(8), *R*_2_^1^(6) and *R*_1_^2^(5) associations. These and additional weak aromatic ring π–π inter­actions [minimum ring-centroid separation = 3.662 (2) Å] give a two-dimensional layered structure.

## Related literature

For guanidinium salts of aromatic acids, see: Parthasarathi *et al.* (1982 ▶); Schürmann *et al.* (1998 ▶); Najafpour *et al.* (2007 ▶); Pereira Silva *et al.* (2007 ▶). For quinaldic acid structures, see: Dobrzyńska & Jerzykiewicz (2004 ▶); Smith *et al.* (2004 ▶, 2007 ▶, 2008*a* ▶,*b* ▶).
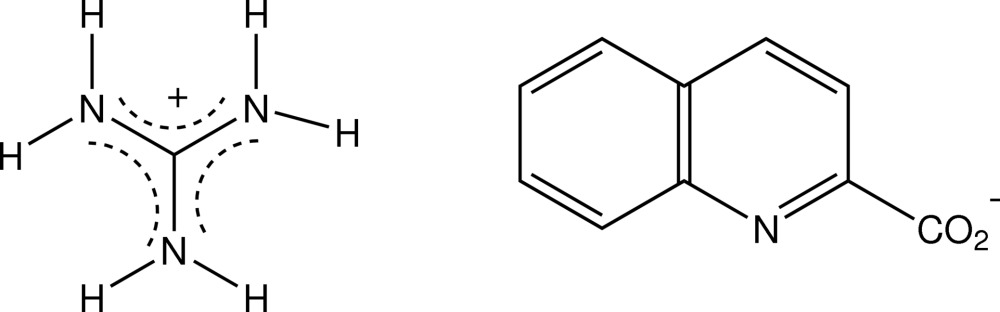


## Experimental

### 

#### Crystal data

CH_6_N_3_^+^·C_10_H_6_NO_2_^−^
*M* *_r_* = 232.25
Monoclinic, 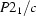

*a* = 7.4318 (3) Å
*b* = 42.2105 (18) Å
*c* = 7.3035 (4) Å
β = 94.045 (4)°
*V* = 2285.40 (18) Å^3^
*Z* = 8
Mo *K*α radiation
μ = 0.10 mm^−1^
*T* = 297 K
0.35 × 0.20 × 0.18 mm

#### Data collection

Oxford Diffraction Gemini-S Ultra CCD-detector diffractometer
Absorption correction: multi-scan (**SADABS**; Sheldrick, 1996 ▶) *T*_min_ = 0.740, *T*_max_ = 0.870
10626 measured reflections
3981 independent reflections
2931 reflections with *I* > 2σ(*I*)
*R*_int_ = 0.035

#### Refinement

*R*[*F* ^2^ > 2σ(*F* ^2^)] = 0.069
*wR*(*F* ^2^) = 0.162
*S* = 1.04
3981 reflections
355 parameters
H atoms treated by a mixture of independent and constrained refinement
Δρ_max_ = 0.15 e Å^−3^
Δρ_min_ = −0.18 e Å^−3^

### 

Data collection: *CrysAlis CCD* (Oxford Diffraction, 2008 ▶); cell refinement: *CrysAlis RED* (Oxford Diffraction, 2008 ▶); data reduction: *CrysAlis RED*; program(s) used to solve structure: *SIR92* (Altomare *et al.*, 1994 ▶); program(s) used to refine structure: *SHELXL97* (Sheldrick, 2008 ▶) within *WinGX* (Farrugia, 1999 ▶); molecular graphics: *PLATON* (Spek, 2009 ▶); software used to prepare material for publication: *PLATON*.

## Supplementary Material

Crystal structure: contains datablocks global, I. DOI: 10.1107/S1600536809049733/wn2367sup1.cif

Structure factors: contains datablocks I. DOI: 10.1107/S1600536809049733/wn2367Isup2.hkl

Additional supplementary materials:  crystallographic information; 3D view; checkCIF report

## Figures and Tables

**Table 1:** Hydrogen-bond geometry (Å, °)

*D*—H⋯*A*	*D*—H	H⋯*A*	*D*⋯*A*	*D*—H⋯*A*
N1*C*—H11*C*⋯O21*B*	0.82 (4)	2.12 (4)	2.937 (5)	176 (3)
N1*C*—H12*C*⋯O21*A* ^i^	0.92 (4)	2.03 (4)	2.852 (5)	149 (3)
N1*D*—H11*D*⋯O22*A*	0.87 (4)	2.04 (4)	2.902 (4)	177 (4)
N1*D*—H12*D*⋯O21*B* ^ii^	0.85 (5)	2.47 (5)	3.312 (5)	173 (4)
N2*C*—H21*C*⋯O22*A*	0.94 (4)	1.87 (4)	2.784 (4)	166 (4)
N2*C*—H22*C*⋯O21*A* ^i^	0.91 (4)	2.57 (4)	3.163 (5)	124 (4)
N2*C*—H22*C*⋯N1*A* ^i^	0.91 (4)	2.08 (5)	2.964 (4)	165 (4)
N2*D*—H21*D*⋯O21*B* ^iii^	0.84 (4)	2.11 (4)	2.899 (5)	155 (3)
N2*D*—H22*D*⋯O21*A*	0.89 (4)	2.01 (5)	2.890 (4)	173 (4)
N3*C*—H31*C*⋯O22*B*	0.93 (5)	1.96 (5)	2.891 (4)	173 (4)
N3*C*—H32*C*⋯O22*A*	0.84 (4)	2.57 (5)	3.216 (5)	135 (4)
N3*D*—H31*D*⋯O21*B* ^iii^	0.93 (4)	2.60 (4)	3.268 (5)	130 (3)
N3*D*—H31*D*⋯N1*B* ^iii^	0.93 (4)	2.12 (4)	3.000 (4)	159 (3)
N3*D*—H32*D*⋯O22*B* ^ii^	0.93 (4)	1.95 (4)	2.826 (4)	157 (4)

Symmetry codes: (i) 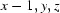; (ii) 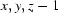; (iii) 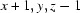.

**Fig. 1.**
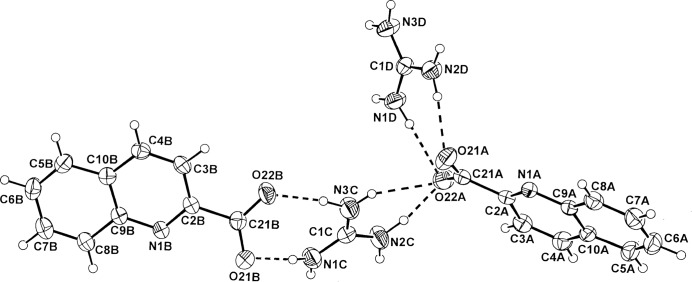
Molecular configuration and atom naming scheme for the two guanidinium cations (C and D) and the two quinoline-2-carboxylate anions (A and B) in the asymmetric unit. Inter-species hydrogen bonds are shown as dashed lines. Displacement ellipsoids are drawn at the 40% probability level.

**Fig. 2.**
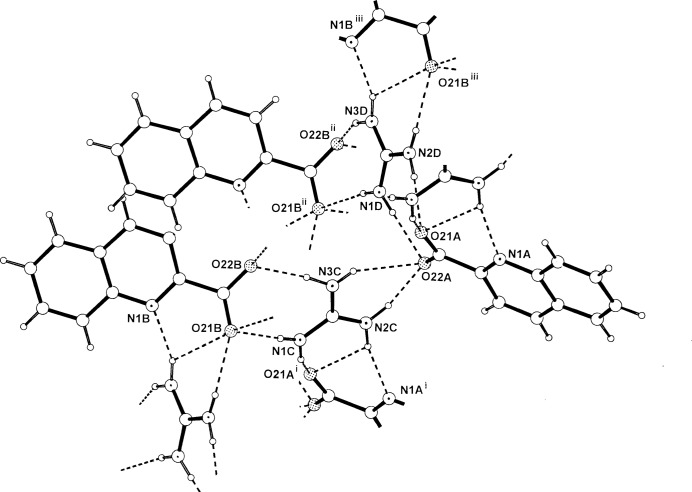
The hydrogen-bonding extensions of the asymmetric unit, showing hydrogen-bonds as dashed lines. For symmetry codes, see Table 1.

**Fig. 3.**
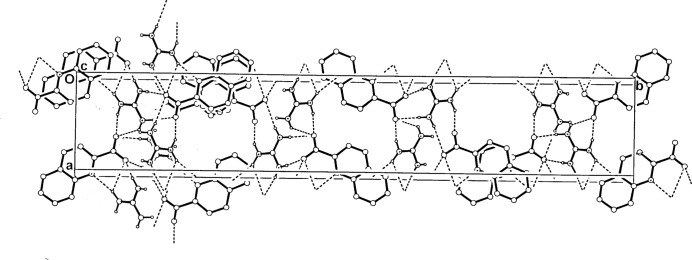
The two-dimensional hydrogen-bonded layered structure, viewed down the c axial direction, showing also quinoline ring overlap. Non-interactive hydrogen atoms are omitted. For symmetry codes, see Table 1.

## supplementary crystallographic information

### Comment

The guanidinium salts of aromatic and heteroaromatic carboxylic acids have proved to be a particularly useful means of generating stable hydrogen-bonded supramolecular framework structures. Three-dimensional structures are most common, largely the result of the interactive efficiency of the symmetrical guanidinium cation in commonly forming cyclic *R*_2_^2^(8) hydrogen-bonding associations with carboxylate-O acceptors. Among the known structures are the guanidinium salts with the aromatic monocarboxylic acids, 4-chloro-3-nitrobenzoic acid (a monohydrate) (Najafpour *et al.*, 2007), and the anhydrous salts with benzoic acid (Pereira Silva *et al.*, 2007), 4-nitrobenzoic acid (Schürmann *et al.*, 1998), 3,5-dinitrobenzoic acid (Smith *et al.*, 2007) and 4-amino-3,5,6-trichloropicolinic acid (Parthasarathi *et al.*, 1982).

Our 1:1 stoichiometric reaction of quinoline-2-carboxylic acid (quinaldic acid) in 50% 2-propanol-water gave large chemically stable crystals of the title compound, anhydrous guanidinium quinoline-2-carboxylate, CH_6_N_3_^+^ C_10_H_6_NO_2_^-^ and the structure is reported here. Quinaldic acid in the solid state exists as a zwitterionic hydrogen-bonded dimer (Dobrzyńska & Jerzykiewicz, 2004) and is commonly found in that form as an adduct species in some proton-transfer compounds where it acts as a Lewis base rather than an acid. Examples are the 1/1/1 quinolinium salt adducts with 5-sulfosalicylic acid (Smith *et al.*, 2004), picrylsulfonic acid (Smith *et al.*, 2008*a*) and 4,5-dichlorophthalic acid (Smith *et al.*, 2008*b*).

In the structure of the title compound the asymmetric unit contains two guanidinium cations (*C* and *D*) and two quinoline-2-carboxylate anions (*A* and *B*) (Fig. 1). The H atom donors of the two cations form similar cyclic hydrogen-bonding interactions with carboxylate O and quinoline N acceptors (Table 1) (Fig. 2), both pairs having two guanidinium-N–H, N'–H'···O associations [graph set *R*_2_^1^(6)] and one N–H, N'–H'···O,*O*' association [*R*_2_^2^(8)]. In addition, each has an *R*_1_^2^(5) guanidinium *N*–*H*···*N*,*O*_quinoline-carboxyl_ association. A two-dimensional layered structure is generated (Fig. 3), in which some aromatic ring overlap down the *c* cell direction gives weak π–π interactions [minimum ring centroid separation,for the six-membered ring N1*B*–C5*B*, 3.662 (2) Å]. The quinoline-2-carboxylate cations are conformationally similar with only minor differences in the N1–C2–C21–O22 torsion angles [170.3 (3)° (*A*), 163.6 (3)° (*B*)].

### Experimental

The title compound was synthesized by heating together under reflux for 10 minutes, 1 mmol quantities of quinoline-2-carboxylic acid and guanidine carbonate in 50 ml of 50% aqueous propan-2-ol. After concentration to *ca* 30 ml, partial room temperature evaporation of the hot-filtered solution gave colourless prisms [m.p. 543–544 K].

### Refinement

Hydrogen atoms involved in hydrogen-bonding interactions were located by difference methods and their positional and isotropic displacement parameters were refined. The aromatic H atoms were included in the refinement in calculated positions (C—H = 0.93 Å) using a riding model approximation, with *U*_iso_(H) = 1.2*U*_eq_(C).

### Crystal data

**Table:**

CH_6_N_3_^+^·C_10_H_6_NO_2_^−^	*F*(000) = 976
*M**_r_* = 232.25	*D*_x_ = 1.350 Mg m^−^^3^
Monoclinic, *P*2_1_/*c*	Melting point = 543–544 K
Hall symbol: -P 2ybc	Mo *K*α radiation, λ = 0.71073 Å
*a* = 7.4318 (3) Å	Cell parameters from 4234 reflections
*b* = 42.2105 (18) Å	θ = 2.9–28.8°
*c* = 7.3035 (4) Å	µ = 0.10 mm^−^^1^
β = 94.045 (4)°	*T* = 297 K
*V* = 2285.40 (18) Å^3^	Prism, colourless
*Z* = 8	0.35 × 0.20 × 0.18 mm

### Data collection

**Table:**

Oxford Diffraction Gemini-S Ultra CCD-detector diffractometer	3981 independent reflections
Radiation source: Enhance (Mo) X-ray source	2931 reflections with *I* > 2σ(*I*)
graphite	*R*_int_ = 0.035
ω scans	θ_max_ = 25.0°, θ_min_ = 2.9°
Absorption correction: multi-scan (*SADABS*; Sheldrick, 1996)	*h* = −8→8
*T*_min_ = 0.740, *T*_max_ = 0.870	*k* = −50→49
10626 measured reflections	*l* = −8→8

### Refinement

**Table:**

Refinement on *F*^2^	Primary atom site location: structure-invariant direct methods
Least-squares matrix: full	Secondary atom site location: difference Fourier map
*R*[*F*^2^ > 2σ(*F*^2^)] = 0.069	Hydrogen site location: inferred from neighbouring sites
*wR*(*F*^2^) = 0.162	H atoms treated by a mixture of independent and constrained refinement
*S* = 1.04	*w* = 1/[σ^2^(*F*_o_^2^) + (0.0614*P*)^2^ + 2.2259*P*] where *P* = (*F*_o_^2^ + 2*F*_c_^2^)/3
3981 reflections	(Δ/σ)_max_ = 0.003
355 parameters	Δρ_max_ = 0.15 e Å^−^^3^
0 restraints	Δρ_min_ = −0.18 e Å^−^^3^

### Special details

**Table:**

Geometry. Bond distances, angles *etc*. have been calculated using the rounded fractional coordinates. All su's are estimated from the variances of the (full) variance-covariance matrix. The cell e.s.d.'s are taken into account in the estimation of distances, angles and torsion angles
Refinement. Refinement of *F*^2^ against ALL reflections. The weighted *R*-factor *wR* and goodness of fit *S* are based on *F*^2^, conventional *R*-factors *R* are based on *F*, with *F* set to zero for negative *F*^2^. The threshold expression of *F*^2^ > σ(*F*^2^) is used only for calculating *R*-factors(gt) *etc*. and is not relevant to the choice of reflections for refinement. *R*-factors based on *F*^2^ are statistically about twice as large as those based on *F*, and *R*- factors based on ALL data will be even larger.

### Fractional atomic coordinates and isotropic or equivalent isotropic displacement parameters (Å^2^)

**Table:**

	*x*	*y*	*z*	*U*_iso_*/*U*_eq_	
O21A	0.8508 (4)	0.09058 (6)	0.2611 (3)	0.0621 (10)	
O22A	0.5996 (3)	0.07308 (6)	0.1162 (4)	0.0645 (10)	
N1A	0.9927 (3)	0.03079 (6)	0.2631 (3)	0.0344 (8)	
C2A	0.8222 (4)	0.03520 (7)	0.2056 (4)	0.0347 (9)	
C3A	0.7048 (4)	0.01015 (8)	0.1538 (4)	0.0432 (11)	
C4A	0.7670 (5)	−0.02006 (8)	0.1610 (4)	0.0470 (11)	
C5A	1.0237 (5)	−0.05662 (8)	0.2320 (5)	0.0526 (14)	
C6A	1.1980 (6)	−0.06067 (9)	0.2937 (5)	0.0607 (14)	
C7A	1.3053 (5)	−0.03505 (10)	0.3470 (5)	0.0573 (14)	
C8A	1.2375 (4)	−0.00486 (8)	0.3367 (4)	0.0463 (11)	
C9A	1.0562 (4)	0.00047 (7)	0.2721 (4)	0.0350 (10)	
C10A	0.9469 (4)	−0.02599 (7)	0.2209 (4)	0.0386 (10)	
C21A	0.7531 (5)	0.06891 (8)	0.1948 (4)	0.0434 (11)	
O21B	0.2113 (4)	0.16103 (6)	0.7760 (4)	0.0595 (9)	
O22B	0.4256 (3)	0.17922 (6)	0.6099 (3)	0.0547 (9)	
N1B	0.0628 (3)	0.22063 (6)	0.7787 (3)	0.0337 (8)	
C2B	0.2260 (4)	0.21671 (7)	0.7234 (4)	0.0331 (9)	
C3B	0.3404 (4)	0.24202 (7)	0.6830 (4)	0.0383 (10)	
C4B	0.2823 (4)	0.27223 (7)	0.7016 (4)	0.0399 (10)	
C5B	0.0359 (5)	0.30838 (8)	0.7780 (4)	0.0467 (11)	
C6B	−0.1343 (5)	0.31222 (9)	0.8310 (5)	0.0521 (12)	
C7B	−0.2392 (5)	0.28594 (9)	0.8685 (5)	0.0500 (11)	
C8B	−0.1739 (4)	0.25609 (8)	0.8528 (4)	0.0417 (11)	
C9B	0.0024 (4)	0.25108 (7)	0.7961 (4)	0.0326 (9)	
C10B	0.1082 (4)	0.27776 (7)	0.7584 (4)	0.0360 (10)	
C21B	0.2913 (4)	0.18309 (8)	0.7021 (4)	0.0389 (10)	
N1C	0.1537 (5)	0.11364 (9)	0.4833 (5)	0.0598 (12)	
N2C	0.2735 (5)	0.08075 (8)	0.2766 (5)	0.0671 (14)	
N3C	0.4496 (5)	0.11943 (9)	0.4200 (5)	0.0622 (12)	
C1C	0.2924 (5)	0.10473 (8)	0.3933 (5)	0.0506 (11)	
N1D	0.6118 (5)	0.13337 (8)	−0.0734 (5)	0.0553 (11)	
N2D	0.9196 (5)	0.13416 (8)	−0.0311 (5)	0.0500 (11)	
N3D	0.7754 (5)	0.17077 (7)	−0.2190 (4)	0.0554 (11)	
C1D	0.7690 (5)	0.14616 (7)	−0.1083 (4)	0.0415 (11)	
H3A	0.58520	0.01430	0.11490	0.0520*	
H4A	0.69050	−0.03680	0.12620	0.0560*	
H5A	0.95370	−0.07410	0.19660	0.0630*	
H6A	1.24640	−0.08100	0.30040	0.0730*	
H7A	1.42460	−0.03830	0.39020	0.0680*	
H8A	1.31110	0.01220	0.37240	0.0560*	
H3B	0.45460	0.23800	0.64390	0.0460*	
H4B	0.35710	0.28910	0.67710	0.0480*	
H5B	0.10530	0.32610	0.75450	0.0560*	
H6B	−0.18120	0.33250	0.84230	0.0630*	
H7B	−0.35550	0.28890	0.90470	0.0600*	
H8B	−0.24530	0.23880	0.87940	0.0500*	
H11C	0.165 (4)	0.1267 (8)	0.567 (5)	0.070 (10)*	
H12C	0.046 (6)	0.1036 (9)	0.454 (5)	0.066 (12)*	
H21C	0.376 (6)	0.0750 (10)	0.217 (6)	0.079 (13)*	
H22C	0.173 (6)	0.0684 (11)	0.272 (6)	0.084 (14)*	
H31C	0.448 (6)	0.1394 (11)	0.475 (6)	0.089 (15)*	
H32C	0.540 (6)	0.1136 (11)	0.367 (6)	0.081 (15)*	
H11D	0.611 (5)	0.1156 (10)	−0.014 (5)	0.068 (13)*	
H12D	0.514 (6)	0.1421 (11)	−0.114 (7)	0.091 (17)*	
H21D	1.022 (5)	0.1409 (8)	−0.055 (5)	0.064 (10)*	
H22D	0.908 (6)	0.1206 (11)	0.060 (6)	0.083 (15)*	
H31D	0.882 (5)	0.1819 (9)	−0.227 (5)	0.060 (11)*	
H32D	0.670 (6)	0.1793 (10)	−0.273 (6)	0.074 (13)*	

### Atomic displacement parameters (Å^2^)

**Table:**

	*U*^11^	*U*^22^	*U*^33^	*U*^12^	*U*^13^	*U*^23^
O21A	0.087 (2)	0.0395 (15)	0.0588 (15)	0.0029 (13)	−0.0028 (14)	0.0009 (11)
O22A	0.0422 (15)	0.0608 (17)	0.092 (2)	0.0122 (12)	0.0149 (14)	0.0230 (14)
N1A	0.0365 (15)	0.0328 (14)	0.0346 (13)	−0.0010 (11)	0.0068 (11)	0.0000 (10)
C2A	0.0352 (17)	0.0373 (17)	0.0327 (15)	−0.0018 (13)	0.0111 (13)	0.0042 (12)
C3A	0.0356 (18)	0.051 (2)	0.0428 (18)	−0.0015 (15)	0.0006 (14)	0.0001 (15)
C4A	0.053 (2)	0.043 (2)	0.0449 (19)	−0.0100 (16)	0.0020 (16)	−0.0061 (15)
C5A	0.076 (3)	0.038 (2)	0.0444 (19)	0.0035 (18)	0.0089 (18)	−0.0019 (15)
C6A	0.085 (3)	0.048 (2)	0.050 (2)	0.021 (2)	0.011 (2)	0.0060 (17)
C7A	0.055 (2)	0.070 (3)	0.047 (2)	0.021 (2)	0.0044 (17)	0.0106 (18)
C8A	0.044 (2)	0.053 (2)	0.0422 (18)	0.0029 (16)	0.0050 (15)	0.0034 (15)
C9A	0.0405 (18)	0.0378 (18)	0.0275 (15)	0.0017 (14)	0.0073 (13)	0.0031 (12)
C10A	0.052 (2)	0.0339 (18)	0.0306 (15)	−0.0008 (14)	0.0090 (14)	−0.0004 (12)
C21A	0.046 (2)	0.044 (2)	0.0421 (18)	0.0062 (16)	0.0173 (15)	0.0097 (15)
O21B	0.0687 (17)	0.0414 (14)	0.0712 (16)	0.0055 (13)	0.0246 (13)	0.0069 (12)
O22B	0.0368 (13)	0.0508 (15)	0.0783 (17)	0.0036 (11)	0.0159 (12)	−0.0133 (12)
N1B	0.0309 (14)	0.0378 (15)	0.0322 (13)	0.0010 (11)	0.0002 (10)	−0.0030 (10)
C2B	0.0286 (16)	0.0401 (17)	0.0304 (15)	0.0018 (13)	−0.0002 (12)	−0.0051 (12)
C3B	0.0292 (16)	0.048 (2)	0.0376 (16)	−0.0003 (14)	0.0027 (13)	−0.0049 (14)
C4B	0.0376 (18)	0.0414 (19)	0.0407 (17)	−0.0082 (14)	0.0023 (14)	−0.0013 (13)
C5B	0.055 (2)	0.0376 (19)	0.0469 (19)	0.0029 (16)	0.0004 (16)	0.0021 (14)
C6B	0.059 (2)	0.044 (2)	0.053 (2)	0.0182 (18)	0.0010 (17)	−0.0025 (16)
C7B	0.043 (2)	0.061 (2)	0.0462 (19)	0.0124 (17)	0.0039 (15)	−0.0062 (16)
C8B	0.0366 (18)	0.048 (2)	0.0406 (18)	0.0015 (15)	0.0027 (14)	−0.0039 (14)
C9B	0.0301 (16)	0.0402 (18)	0.0268 (14)	0.0026 (13)	−0.0032 (12)	−0.0033 (12)
C10B	0.0382 (17)	0.0405 (18)	0.0286 (15)	0.0010 (14)	−0.0028 (12)	−0.0013 (12)
C21B	0.0332 (17)	0.0404 (19)	0.0425 (17)	0.0017 (14)	−0.0007 (14)	−0.0025 (14)
N1C	0.060 (2)	0.054 (2)	0.068 (2)	−0.0068 (18)	0.0225 (18)	−0.0130 (17)
N2C	0.055 (2)	0.052 (2)	0.098 (3)	−0.0159 (17)	0.032 (2)	−0.0300 (18)
N3C	0.055 (2)	0.050 (2)	0.084 (2)	−0.0109 (17)	0.0209 (18)	−0.0184 (17)
C1C	0.056 (2)	0.0367 (19)	0.061 (2)	−0.0033 (17)	0.0166 (18)	0.0032 (16)
N1D	0.050 (2)	0.0452 (19)	0.071 (2)	−0.0032 (16)	0.0071 (16)	0.0158 (16)
N2D	0.047 (2)	0.0478 (19)	0.0550 (19)	−0.0004 (16)	0.0023 (15)	0.0055 (15)
N3D	0.047 (2)	0.0462 (19)	0.072 (2)	−0.0081 (16)	−0.0022 (16)	0.0205 (15)
C1D	0.046 (2)	0.0344 (18)	0.0445 (18)	−0.0033 (15)	0.0051 (15)	−0.0031 (14)

### Geometric parameters (Å, °)

**Table:**

O21A—C21A	1.245 (4)	C4A—C10A	1.400 (5)
O22A—C21A	1.253 (4)	C5A—C6A	1.352 (6)
O21B—C21B	1.248 (4)	C5A—C10A	1.413 (5)
O22B—C21B	1.253 (4)	C6A—C7A	1.383 (6)
N1A—C2A	1.320 (4)	C7A—C8A	1.371 (5)
N1A—C9A	1.364 (4)	C8A—C9A	1.414 (4)
N1B—C2B	1.316 (4)	C9A—C10A	1.416 (4)
N1B—C9B	1.370 (4)	C3A—H3A	0.9300
N1C—C1C	1.316 (5)	C4A—H4A	0.9300
N2C—C1C	1.324 (5)	C5A—H5A	0.9300
N3C—C1C	1.325 (5)	C6A—H6A	0.9300
N1C—H11C	0.82 (4)	C7A—H7A	0.9300
N1C—H12C	0.92 (4)	C8A—H8A	0.9300
N2C—H21C	0.94 (4)	C2B—C21B	1.511 (4)
N2C—H22C	0.91 (4)	C2B—C3B	1.410 (4)
N3C—H32C	0.84 (4)	C3B—C4B	1.356 (4)
N3C—H31C	0.93 (5)	C4B—C10B	1.406 (4)
N1D—C1D	1.328 (5)	C5B—C10B	1.411 (5)
N2D—C1D	1.318 (5)	C5B—C6B	1.358 (5)
N3D—C1D	1.319 (4)	C6B—C7B	1.394 (5)
N1D—H11D	0.87 (4)	C7B—C8B	1.358 (5)
N1D—H12D	0.85 (5)	C8B—C9B	1.418 (4)
N2D—H21D	0.84 (4)	C9B—C10B	1.412 (4)
N2D—H22D	0.89 (4)	C3B—H3B	0.9300
N3D—H31D	0.93 (4)	C4B—H4B	0.9300
N3D—H32D	0.93 (4)	C5B—H5B	0.9300
C2A—C21A	1.513 (5)	C6B—H6B	0.9300
C2A—C3A	1.406 (4)	C7B—H7B	0.9300
C3A—C4A	1.356 (5)	C8B—H8B	0.9300
			
O21A···N1A	2.735 (4)	C10B···C2B^v^	3.455 (4)
O21A···N1C^i^	2.852 (5)	C10B···C3B^v^	3.541 (4)
O21A···N2C^i^	3.163 (5)	C21B···C5B^xi^	3.535 (4)
O21A···N2D	2.890 (4)	C1D···H6A^x^	3.0900
O21A···C1C^i^	3.408 (5)	C2A···H22C^i^	2.97 (5)
O21B···N1C	2.937 (5)	C2B···H31D^ii^	2.99 (4)
O21B···N2D^ii^	2.899 (5)	C3A···H3A^xiii^	2.9900
O21B···N1B	2.748 (4)	C4B···H7B^i^	3.0600
O21B···N3D^ii^	3.268 (5)	C9A···H22C^i^	3.00 (5)
O22A···N3C	3.216 (5)	C9B···H31D^ii^	3.06 (4)
O22A···N1D	2.902 (4)	C21A···H21C	2.83 (4)
O22A···N2C	2.784 (4)	C21A···H32C	2.82 (5)
O22B···N1D^iii^	3.250 (4)	C21A···H11D	2.66 (4)
O22B···N3C	2.891 (4)	C21A···H22D	2.69 (5)
O22B···N3D^iii^	2.826 (4)	C21B···H11C	2.72 (3)
O21A···H12C^i^	2.03 (4)	C21B···H31C	2.79 (5)
O21A···H11D	2.80 (4)	C21B···H32D^iii^	2.81 (4)
O21A···H22C^i^	2.57 (4)	C21B···H12D^iii^	2.69 (5)
O21A···H32C	2.67 (5)	H3A···O22A	2.4800
O21A···H22D	2.01 (5)	H3A···C3A^xiii^	2.9900
O21B···H21D^ii^	2.11 (4)	H3A···H3A^xiii^	2.3600
O21B···H31D^ii^	2.60 (4)	H3B···H7B^iv^	2.5800
O21B···H11C	2.12 (4)	H3B···O22B	2.5000
O21B···H12D^iii^	2.47 (5)	H4A···H5A	2.5400
O22A···H3A	2.4800	H4B···H5B	2.5300
O22A···H32C	2.57 (5)	H5A···H4A	2.5400
O22A···H11D	2.04 (4)	H5B···H4B	2.5300
O22A···H21C	1.87 (4)	H5B···N2D^xii^	2.9400
O22B···H12D^iii^	2.60 (5)	H6A···C1D^x^	3.0900
O22B···H7B^iv^	2.6500	H7B···H3B^xii^	2.5800
O22B···H31C	1.96 (5)	H7B···C4B^vi^	3.0600
O22B···H32D^iii^	1.95 (4)	H7B···O22B^xii^	2.6500
O22B···H3B	2.5000	H11C···C21B	2.72 (3)
N1A···O21A	2.735 (4)	H11C···O21B	2.12 (4)
N1A···N2C^i^	2.964 (4)	H11C···H31C	2.32 (5)
N1B···O21B	2.748 (4)	H11D···H22D	2.25 (6)
N1B···N3D^ii^	3.000 (4)	H11D···C21A	2.66 (4)
N1B···C4B^v^	3.403 (4)	H11D···O21A	2.80 (4)
N1C···O21B	2.937 (5)	H11D···O22A	2.04 (4)
N1C···O21A^vi^	2.852 (5)	H12C···H22C	2.25 (6)
N1D···O22A	2.902 (4)	H12C···O21A^vi^	2.03 (4)
N1D···O22B^vii^	3.250 (4)	H12D···O21B^vii^	2.47 (5)
N2C···N1A^vi^	2.964 (4)	H12D···C21B^vii^	2.69 (5)
N2C···O21A^vi^	3.163 (5)	H12D···H32D	2.31 (6)
N2C···O22A	2.784 (4)	H12D···O22B^vii^	2.60 (5)
N2D···C5B^iv^	3.384 (5)	H21C···C21A	2.83 (4)
N2D···O21B^viii^	2.899 (5)	H21C···H32C	2.27 (6)
N2D···O21A	2.890 (4)	H21C···O22A	1.87 (4)
N3C···O22B	2.891 (4)	H21D···O21B^viii^	2.11 (4)
N3C···O22A	3.216 (5)	H21D···H31D	2.34 (5)
N3D···O22B^vii^	2.826 (4)	H22C···C2A^vi^	2.97 (5)
N3D···O21B^viii^	3.268 (5)	H22C···C9A^vi^	3.00 (5)
N3D···N1B^viii^	3.000 (4)	H22C···H12C	2.25 (6)
N1A···H22C^i^	2.08 (5)	H22C···N1A^vi^	2.08 (5)
N1B···H31D^ii^	2.12 (4)	H22C···O21A^vi^	2.57 (4)
N2D···H5B^iv^	2.9400	H22D···O21A	2.01 (5)
C1C···O21A^vi^	3.408 (5)	H22D···C21A	2.69 (5)
C2A···C7A^ix^	3.466 (5)	H22D···H11D	2.25 (6)
C2A···C5A^x^	3.586 (5)	H31C···C21B	2.79 (5)
C2B···C10B^xi^	3.455 (4)	H31C···H11C	2.32 (5)
C2B···C4B^v^	3.519 (4)	H31C···O22B	1.96 (5)
C3B···C4B^xi^	3.563 (4)	H31D···H21D	2.34 (5)
C3B···C10B^xi^	3.541 (4)	H31D···O21B^viii^	2.60 (4)
C4B···C2B^xi^	3.519 (4)	H31D···N1B^viii^	2.12 (4)
C4B···C3B^v^	3.563 (4)	H31D···C9B^viii^	3.06 (4)
C4B···N1B^xi^	3.403 (4)	H31D···C2B^viii^	2.99 (4)
C5A···C2A^x^	3.586 (5)	H32C···C21A	2.82 (5)
C5B···C21B^v^	3.535 (4)	H32C···H21C	2.27 (6)
C5B···N2D^xii^	3.384 (5)	H32C···O22A	2.57 (5)
C7A···C2A^ix^	3.466 (5)	H32C···O21A	2.67 (5)
C8B···C9B^v^	3.419 (4)	H32D···O22B^vii^	1.95 (4)
C9A···C9A^ix^	3.489 (4)	H32D···C21B^vii^	2.81 (4)
C9B···C8B^xi^	3.419 (4)	H32D···H12D	2.31 (6)
			
C2A—N1A—C9A	118.0 (3)	C6A—C5A—H5A	120.00
C2B—N1B—C9B	117.5 (3)	C7A—C6A—H6A	120.00
C1C—N1C—H11C	121 (2)	C5A—C6A—H6A	119.00
C1C—N1C—H12C	117 (3)	C8A—C7A—H7A	120.00
H11C—N1C—H12C	122 (3)	C6A—C7A—H7A	120.00
C1C—N2C—H21C	116 (3)	C7A—C8A—H8A	120.00
C1C—N2C—H22C	121 (3)	C9A—C8A—H8A	120.00
H21C—N2C—H22C	122 (4)	N1B—C2B—C21B	117.4 (3)
C1C—N3C—H31C	117 (3)	C3B—C2B—C21B	119.2 (3)
C1C—N3C—H32C	122 (3)	N1B—C2B—C3B	123.5 (3)
H31C—N3C—H32C	120 (4)	C2B—C3B—C4B	119.4 (3)
C1D—N1D—H11D	119 (2)	C3B—C4B—C10B	119.5 (3)
C1D—N1D—H12D	120 (3)	C6B—C5B—C10B	120.5 (3)
H11D—N1D—H12D	121 (4)	C5B—C6B—C7B	120.4 (3)
C1D—N2D—H22D	116 (3)	C6B—C7B—C8B	120.9 (3)
H21D—N2D—H22D	121 (4)	C7B—C8B—C9B	120.4 (3)
C1D—N2D—H21D	122 (2)	C8B—C9B—C10B	118.5 (3)
C1D—N3D—H31D	120 (2)	N1B—C9B—C8B	118.9 (3)
C1D—N3D—H32D	120 (3)	N1B—C9B—C10B	122.7 (3)
H31D—N3D—H32D	119 (4)	C4B—C10B—C9B	117.5 (3)
N1A—C2A—C21A	117.7 (3)	C5B—C10B—C9B	119.3 (3)
N1A—C2A—C3A	122.9 (3)	C4B—C10B—C5B	123.2 (3)
C3A—C2A—C21A	119.4 (3)	O21B—C21B—C2B	119.3 (3)
C2A—C3A—C4A	119.6 (3)	O21B—C21B—O22B	123.8 (3)
C3A—C4A—C10A	119.7 (3)	O22B—C21B—C2B	116.8 (3)
C6A—C5A—C10A	120.6 (3)	C2B—C3B—H3B	120.00
C5A—C6A—C7A	121.0 (4)	C4B—C3B—H3B	120.00
C6A—C7A—C8A	120.6 (3)	C10B—C4B—H4B	120.00
C7A—C8A—C9A	120.3 (3)	C3B—C4B—H4B	120.00
N1A—C9A—C10A	122.5 (3)	C6B—C5B—H5B	120.00
N1A—C9A—C8A	118.9 (3)	C10B—C5B—H5B	120.00
C8A—C9A—C10A	118.6 (3)	C7B—C6B—H6B	120.00
C5A—C10A—C9A	119.0 (3)	C5B—C6B—H6B	120.00
C4A—C10A—C9A	117.3 (3)	C8B—C7B—H7B	120.00
C4A—C10A—C5A	123.7 (3)	C6B—C7B—H7B	120.00
O21A—C21A—C2A	119.0 (3)	C9B—C8B—H8B	120.00
O21A—C21A—O22A	124.2 (3)	C7B—C8B—H8B	120.00
O22A—C21A—C2A	116.8 (3)	N2C—C1C—N3C	120.3 (4)
C2A—C3A—H3A	120.00	N1C—C1C—N2C	119.3 (4)
C4A—C3A—H3A	120.00	N1C—C1C—N3C	120.4 (3)
C3A—C4A—H4A	120.00	N2D—C1D—N3D	119.9 (3)
C10A—C4A—H4A	120.00	N1D—C1D—N2D	119.6 (3)
C10A—C5A—H5A	120.00	N1D—C1D—N3D	120.5 (3)
			
C9A—N1A—C2A—C3A	0.0 (4)	N1A—C9A—C10A—C5A	179.2 (3)
C9A—N1A—C2A—C21A	−179.6 (2)	C8A—C9A—C10A—C4A	178.7 (3)
C2A—N1A—C9A—C8A	−178.8 (3)	C8A—C9A—C10A—C5A	−1.5 (4)
C2A—N1A—C9A—C10A	0.6 (4)	N1A—C9A—C10A—C4A	−0.6 (4)
C2B—N1B—C9B—C8B	179.1 (3)	N1B—C2B—C3B—C4B	0.2 (5)
C2B—N1B—C9B—C10B	−0.4 (4)	C21B—C2B—C3B—C4B	179.9 (3)
C9B—N1B—C2B—C3B	0.4 (4)	N1B—C2B—C21B—O21B	−16.7 (4)
C9B—N1B—C2B—C21B	−179.3 (2)	N1B—C2B—C21B—O22B	163.6 (3)
C3A—C2A—C21A—O21A	171.5 (3)	C3B—C2B—C21B—O21B	163.6 (3)
N1A—C2A—C21A—O21A	−8.9 (4)	C3B—C2B—C21B—O22B	−16.1 (4)
N1A—C2A—C21A—O22A	170.3 (3)	C2B—C3B—C4B—C10B	−0.9 (4)
N1A—C2A—C3A—C4A	−0.5 (5)	C3B—C4B—C10B—C5B	−178.8 (3)
C21A—C2A—C3A—C4A	179.1 (3)	C3B—C4B—C10B—C9B	0.9 (4)
C3A—C2A—C21A—O22A	−9.4 (4)	C10B—C5B—C6B—C7B	0.6 (5)
C2A—C3A—C4A—C10A	0.4 (4)	C6B—C5B—C10B—C4B	179.1 (3)
C3A—C4A—C10A—C9A	0.1 (4)	C6B—C5B—C10B—C9B	−0.6 (5)
C3A—C4A—C10A—C5A	−179.7 (3)	C5B—C6B—C7B—C8B	−0.1 (5)
C6A—C5A—C10A—C4A	−179.0 (3)	C6B—C7B—C8B—C9B	−0.6 (5)
C6A—C5A—C10A—C9A	1.2 (5)	C7B—C8B—C9B—N1B	−178.9 (3)
C10A—C5A—C6A—C7A	−0.1 (6)	C7B—C8B—C9B—C10B	0.6 (4)
C5A—C6A—C7A—C8A	−0.6 (6)	N1B—C9B—C10B—C4B	−0.2 (4)
C6A—C7A—C8A—C9A	0.3 (5)	N1B—C9B—C10B—C5B	179.5 (3)
C7A—C8A—C9A—N1A	−179.9 (3)	C8B—C9B—C10B—C4B	−179.7 (3)
C7A—C8A—C9A—C10A	0.8 (4)	C8B—C9B—C10B—C5B	0.0 (4)

Symmetry codes: (i) *x*+1, *y*, *z*; (ii) *x*−1, *y*, *z*+1; (iii) *x*, *y*, *z*+1; (iv) *x*+1, −*y*+1/2, *z*−1/2; (v) *x*, −*y*+1/2, *z*+1/2; (vi) *x*−1, *y*, *z*; (vii) *x*, *y*, *z*−1; (viii) *x*+1, *y*, *z*−1; (ix) −*x*+2, −*y*, −*z*+1; (x) −*x*+2, −*y*, −*z*; (xi) *x*, −*y*+1/2, *z*−1/2; (xii) *x*−1, −*y*+1/2, *z*+1/2; (xiii) −*x*+1, −*y*, −*z*.

### Hydrogen-bond geometry (Å, °)

**Table:**

*D*—H···*A*	*D*—H	H···*A*	*D*···*A*	*D*—H···*A*
N1C—H11C···O21B	0.82 (4)	2.12 (4)	2.937 (5)	176 (3)
N1C—H12C···O21A^vi^	0.92 (4)	2.03 (4)	2.852 (5)	149 (3)
N1D—H11D···O22A	0.87 (4)	2.04 (4)	2.902 (4)	177 (4)
N1D—H12D···O21B^vii^	0.85 (5)	2.47 (5)	3.312 (5)	173 (4)
N2C—H21C···O22A	0.94 (4)	1.87 (4)	2.784 (4)	166 (4)
N2C—H22C···O21A^vi^	0.91 (4)	2.57 (4)	3.163 (5)	124 (4)
N2C—H22C···N1A^vi^	0.91 (4)	2.08 (5)	2.964 (4)	165 (4)
N2D—H21D···O21B^viii^	0.84 (4)	2.11 (4)	2.899 (5)	155 (3)
N2D—H22D···O21A	0.89 (4)	2.01 (5)	2.890 (4)	173 (4)
N3C—H31C···O22B	0.93 (5)	1.96 (5)	2.891 (4)	173 (4)
N3C—H32C···O22A	0.84 (4)	2.57 (5)	3.216 (5)	135 (4)
N3D—H31D···O21B^viii^	0.93 (4)	2.60 (4)	3.268 (5)	130 (3)
N3D—H31D···N1B^viii^	0.93 (4)	2.12 (4)	3.000 (4)	159 (3)
N3D—H32D···O22B^vii^	0.93 (4)	1.95 (4)	2.826 (4)	157 (4)

Symmetry codes: (vi) *x*−1, *y*, *z*; (vii) *x*, *y*, *z*−1; (viii) *x*+1, *y*, *z*−1.
